# Supplementary material for: Efficacy of Flavonoids on Animal Models of Polycystic Ovary Syndrome: A Systematic Review and Meta-Analysis
Source: Nutrients. 2022 Oct 4;14(19):4128. doi: 10.3390/nu14194128 (PMC9571610; doi:10.3390/nu14194128)
Supplement: Supplementary file 1 [file nutrients-14-04128-s001.zip › nutrients-1933156-supplementary.pdf]

SUPPLEMENTARY MATERIALS

Supplementary Figure 1

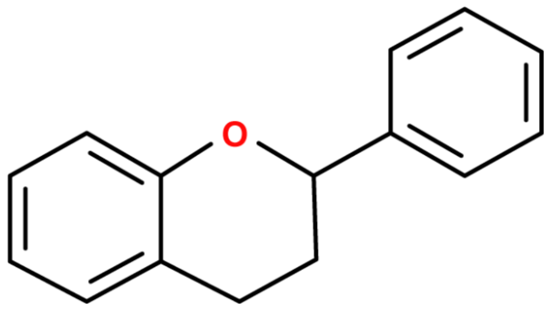

Figure S1 Basic chemical structure of flavonoids.

Supplementary Figure 2

	Allocation concealment	Baseline characteristics	Blinding of outcome assessors	Blinding of trial caregivers	Incomplete outcome data	Other sources	Random housing	Random outcome assessment	Selective outcome reporting	Sequence generation
Bulsara J 2022	?	+	?	?	+	?	?	?	+	?
Darabi P 2019	?	+	?	?	+	-	?	?	+	?
Hong G 2020	?	+	?	?	+	-	?	?	?	?
Hu T 2017	?	+	?	?	?	+	?	?	?	?
Jahan S 2016	?	+	?	?	+	-	?	?	+	?
Jahan S 2018	?	+	+	?	+	-	?	?	+	+
Liyanage GS 2021	?	+	+	?	+	-	?	?	+	?
Ma XH 2021	?	+	?	?	+	?	?	?	+	+
Mahmoud AA 2022	?	+	?	?	+	?	?	?	+	?
Manzar N 2020	?	?	?	?	+	-	?	?	+	?
Mihanfar A 2021	?	+	?	?	+	-	?	?	+	?
Moshfegh F 2022	?	+	?	?	+	+	?	?	+	+
Peng FX 2022	?	+	?	?	+	-	?	?	+	?
Rajan RK 2017	?	+	?	?	?	+	?	?	?	?
Shah KN 2015	?	+	?	?	+	-	?	?	+	?
Wang W 2019	?	+	?	?	+	?	?	?	+	?
Yu J 2019	?	+	?	?	+	?	?	?	+	?
Zheng SY 2021	?	+	?	?	+	?	?	?	+	+

Figure S2 Evaluation of quality for each study.

Supplementary Figure 3

Study	Experimental Total	Experimental Mean	Experimental SD	Control Total	Control Mean	Control SD	SMD	95%-CI	Weight
Mahmoud AA 2022	7	20.05	3.3600	7	21.95	5.8700	-0.37	[-1.43; 0.69]	10.3%
Moshfegh F 2022	12	5.26	1.0700	12	2.84	1.1100	2.14	[1.11; 3.18]	10.3%
Liyanage GS 2021	8	36.44	13.5500	8	35.42	11.6000	0.08	[-0.90; 1.06]	10.4%
Ma XH 2021	5	5.91	1.0900	5	3.09	0.7000	2.78	[0.80; 4.75]	9.0%
Zheng SY 2021	10	8.16	0.0700	10	7.48	0.2500	3.55	[2.05; 5.05]	9.7%
Manzar N 2020	9	4.67	0.3200	9	4.37	0.3200	0.89	[-0.09; 1.87]	10.4%
Hong G 2020	10	32.70	3.2100	10	35.03	2.9770	-0.72	[-1.63; 0.19]	10.4%
Wang W 2019	10	4.95	0.1900	10	7.03	0.4800	-5.46	[-7.53; -3.39]	8.8%
Yu J 2019	15	12.10	1.0800	15	11.13	1.0900	0.87	[0.12; 1.62]	10.6%
Darabi P 2019	8	6.67	1.1300	8	3.90	0.0000			0.0%
Hu T 2017	5	1.94	0.1000	5	1.96	0.1100	-0.17	[-1.41; 1.07]	10.1%
**Random effects model**	**99**			**99**			**0.39**	**[-1.02; 1.81]**	**100.0%**

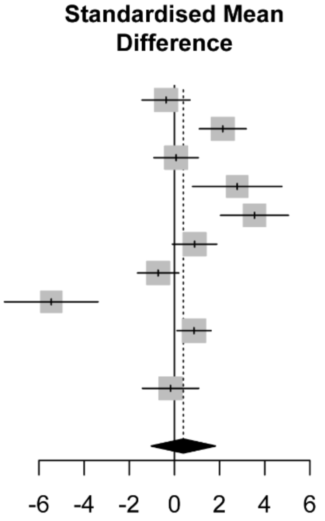

Heterogeneity: $I^2 = 88\%$, $\tau^2 = 4.7872$, $p < 0.01$
Overall test: $z = 0.55$ ($p = 0.58$)

Figure S3 Forest plot of flavonoids for FSH.

Supplementary Figure 4

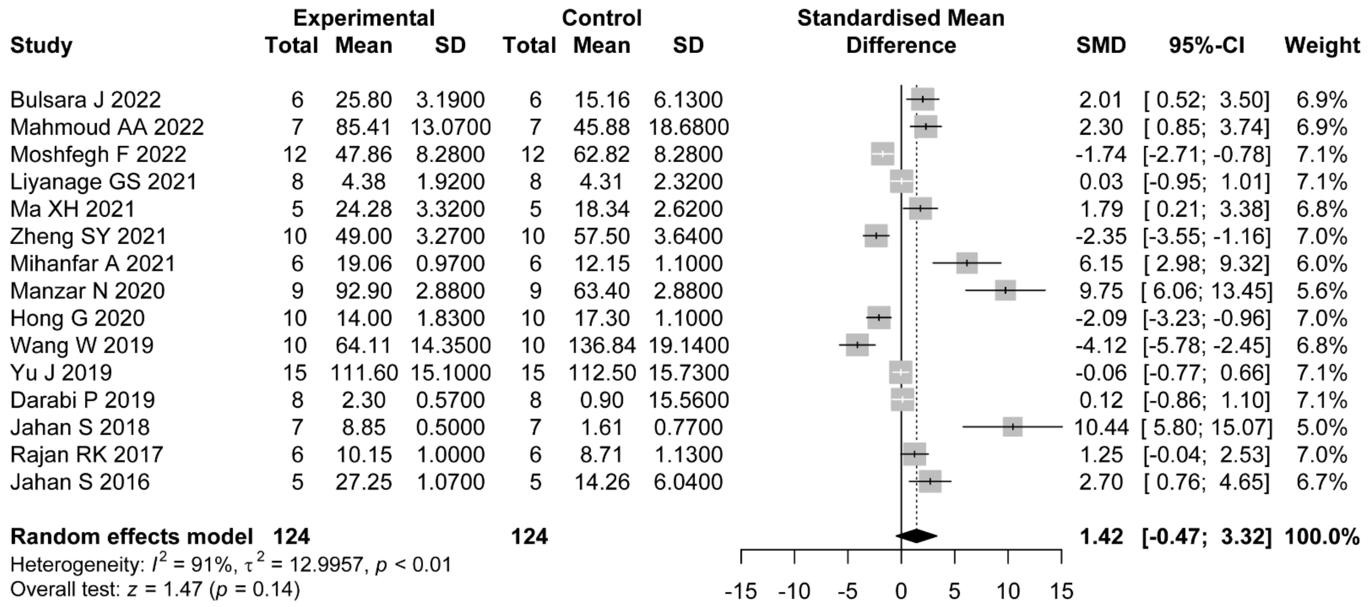

Figure S4 Forest plot of flavonoids for estradiol.

Supplementary Figure 5

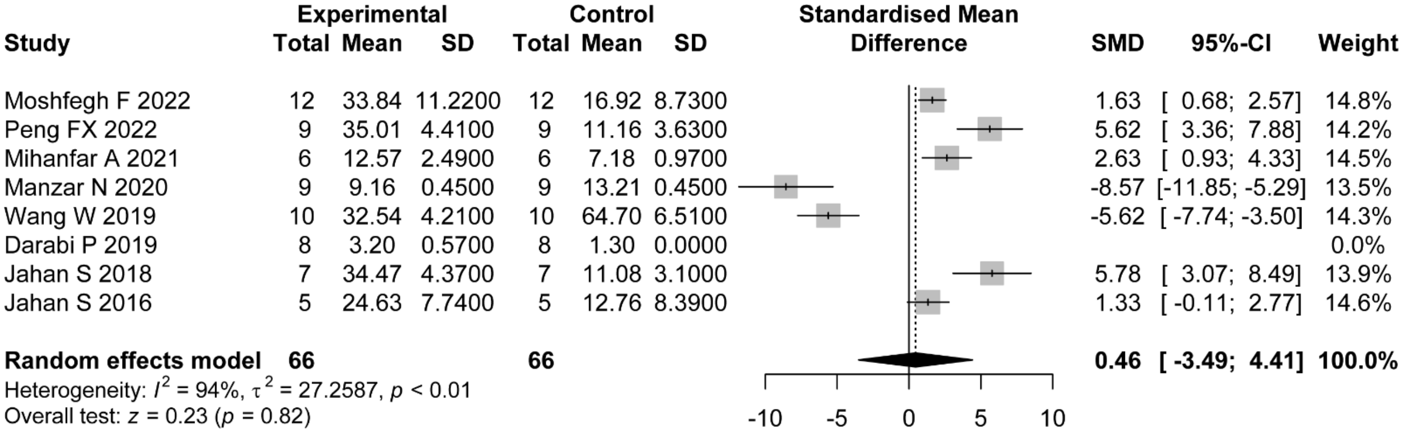

Figure S5 Forest plot of flavonoids for progesterone.

Supplementary Figure 6

(A)

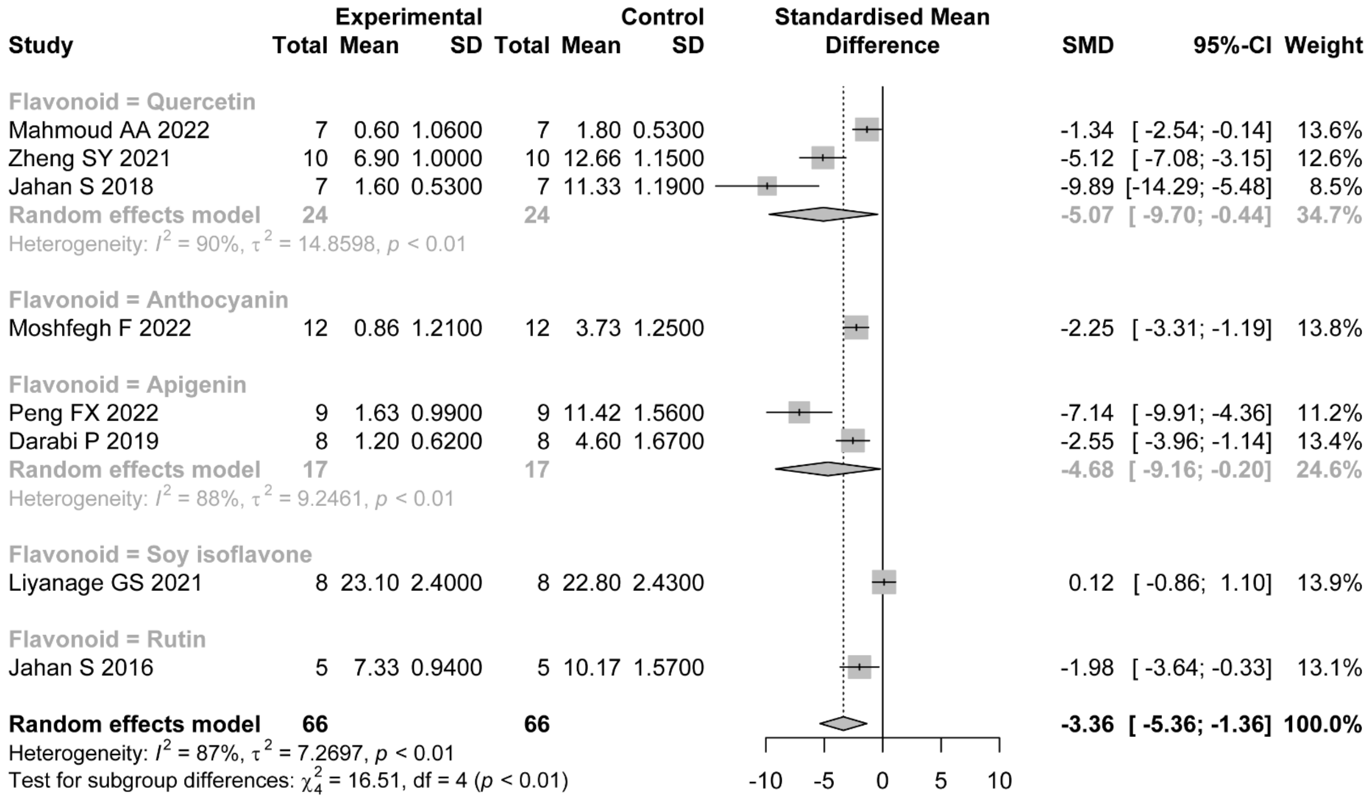

(B)

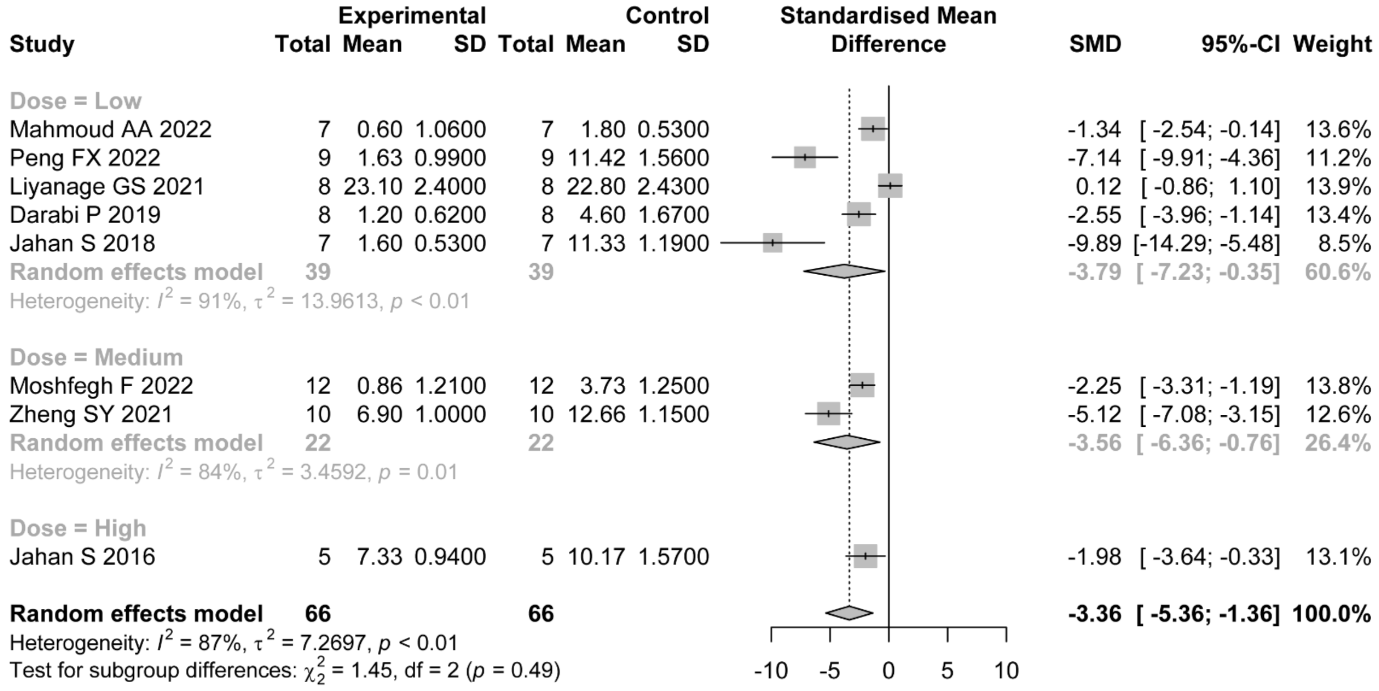

(C)

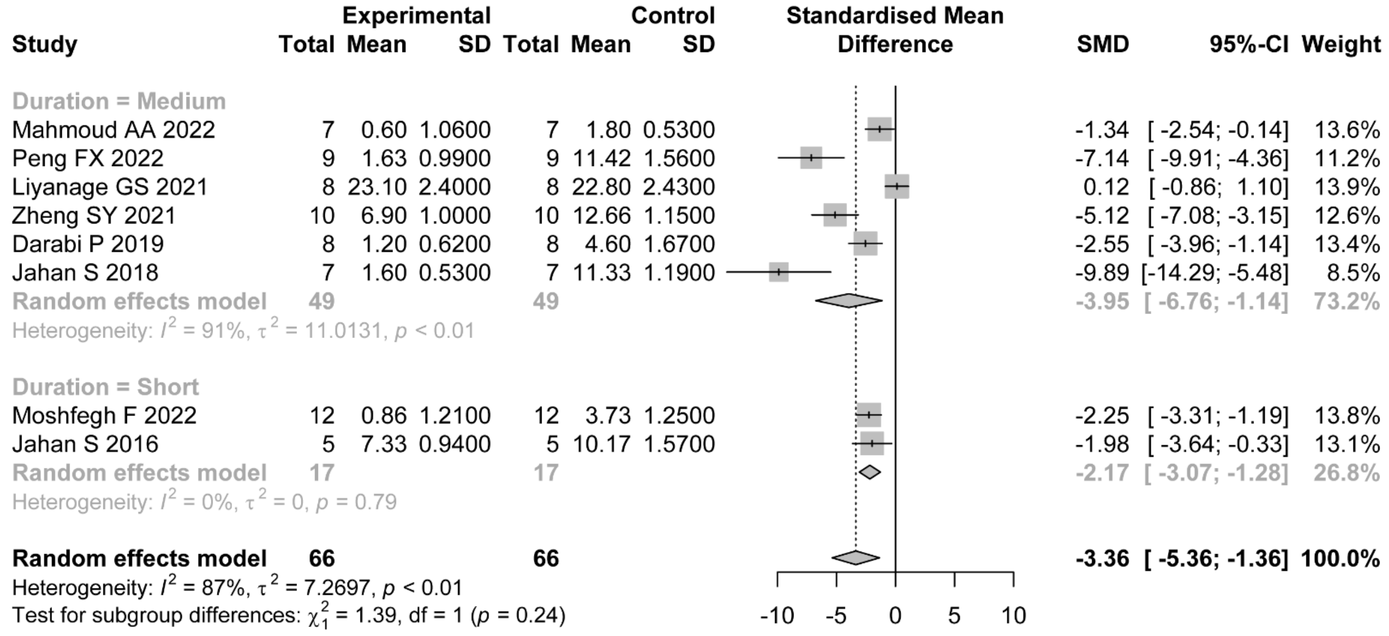

(D)

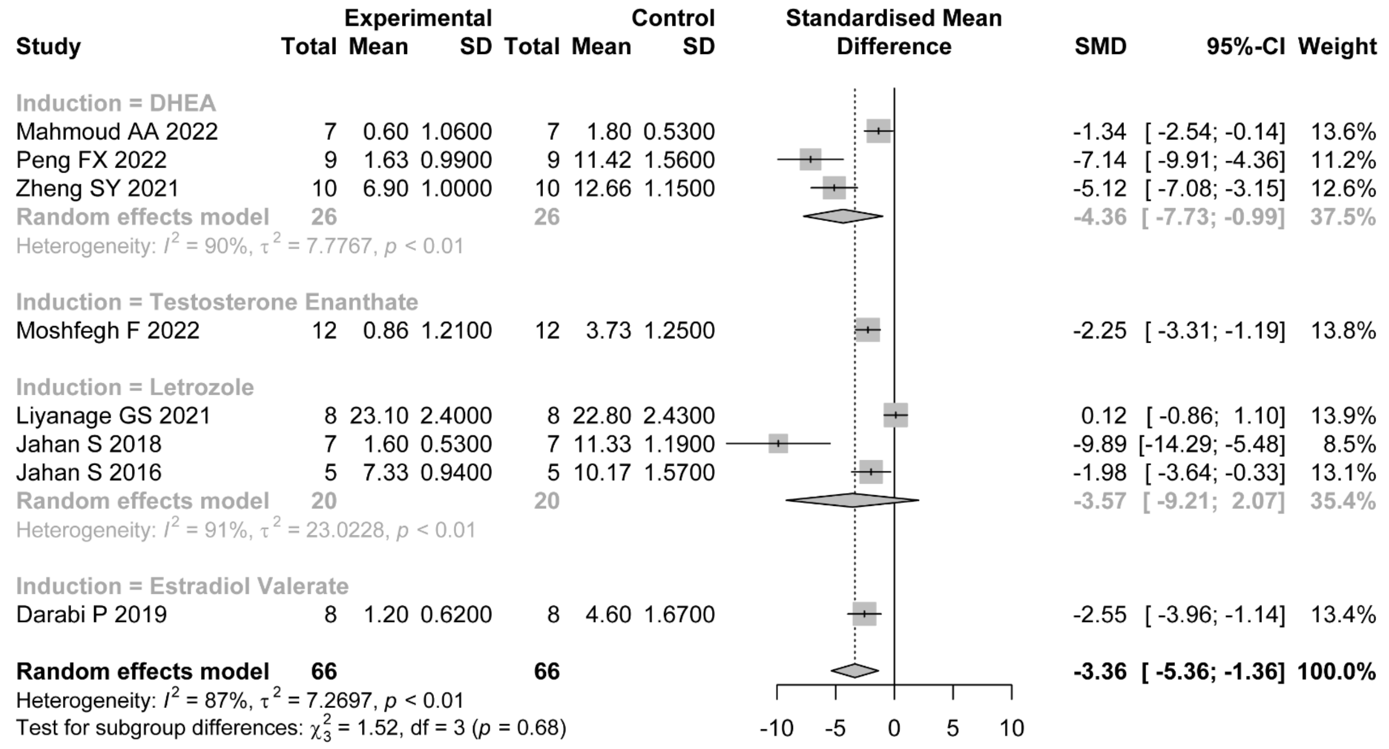

Figure S6 Subgroup analyses of the count of cystic follicles with **(A)** type of flavonoids, **(B)** dose, **(C)** duration of administration, and **(D)** PCOS induction drug.

Supplementary Figure 7

(A)

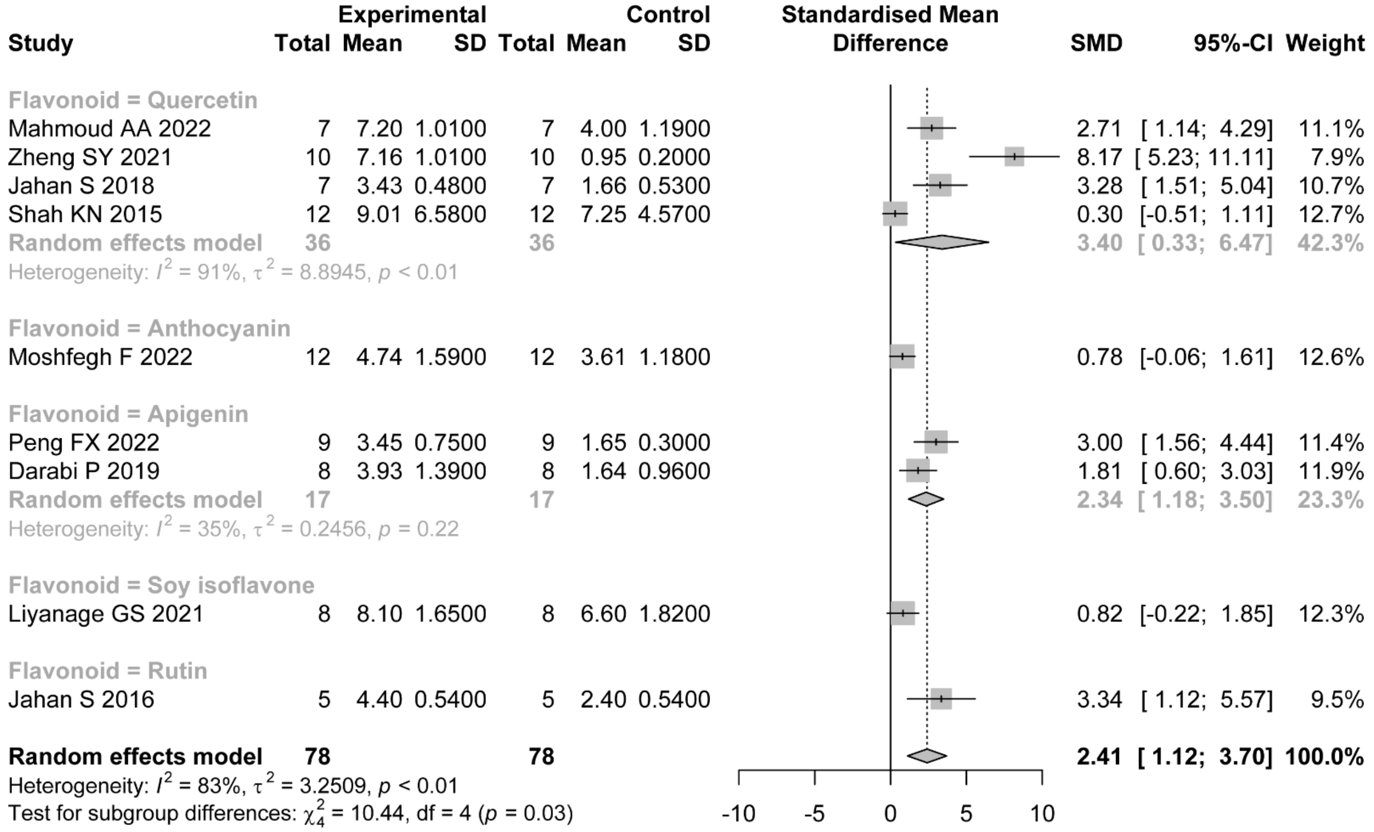

(B)

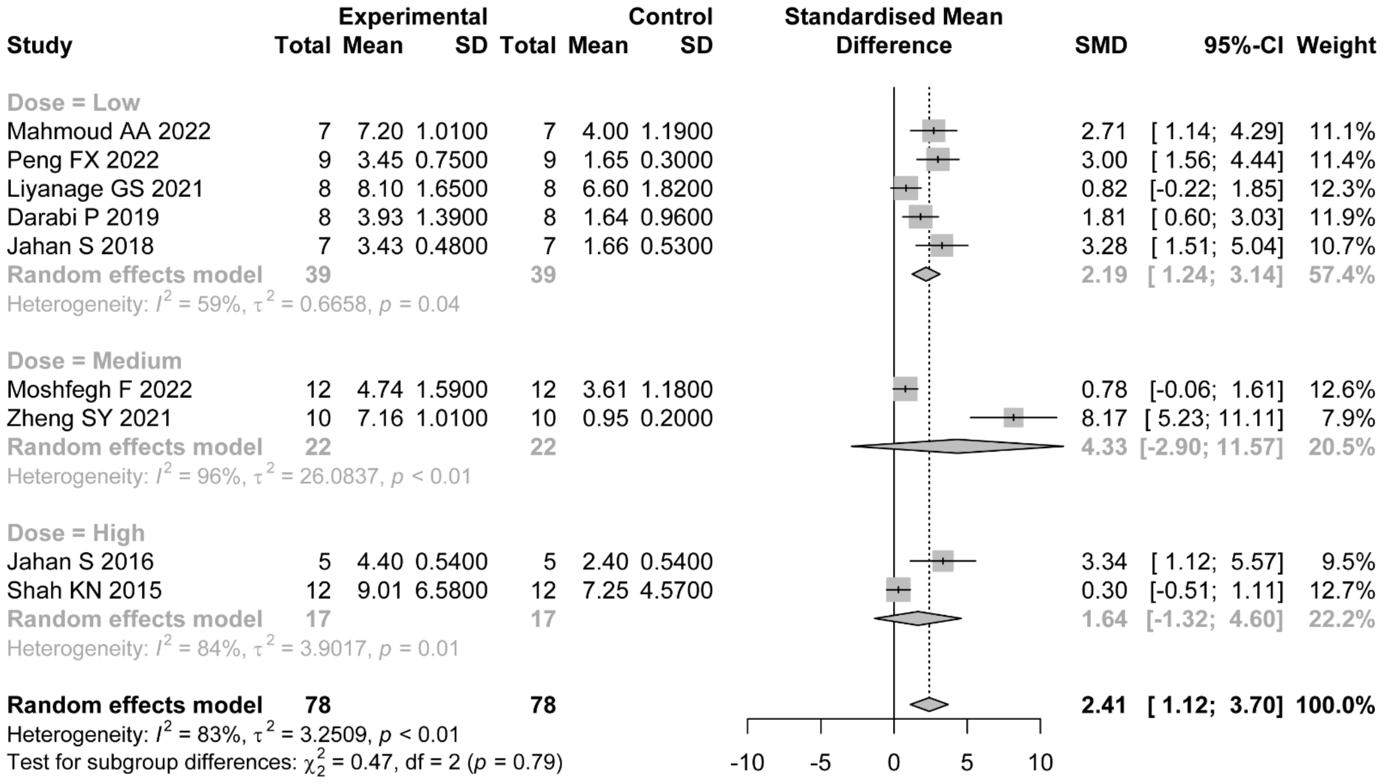

(C)

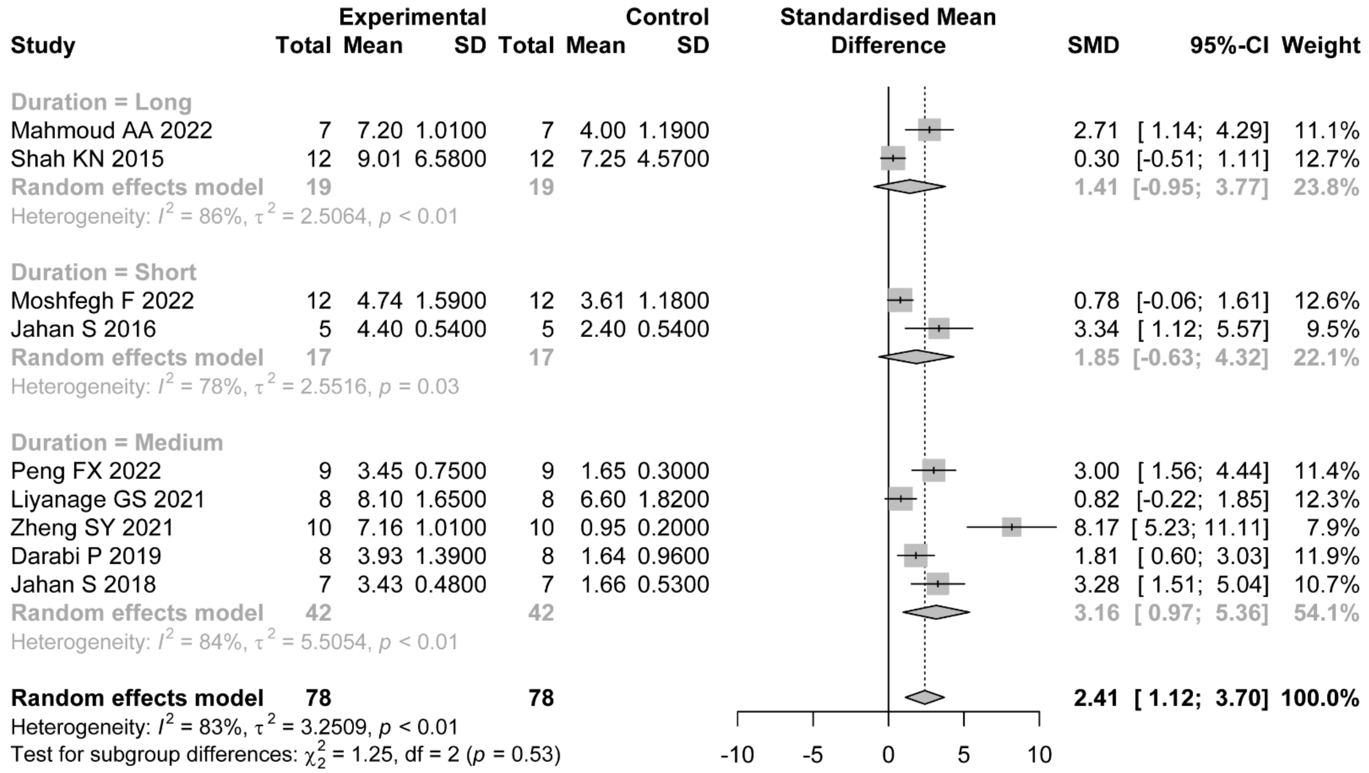

(D)

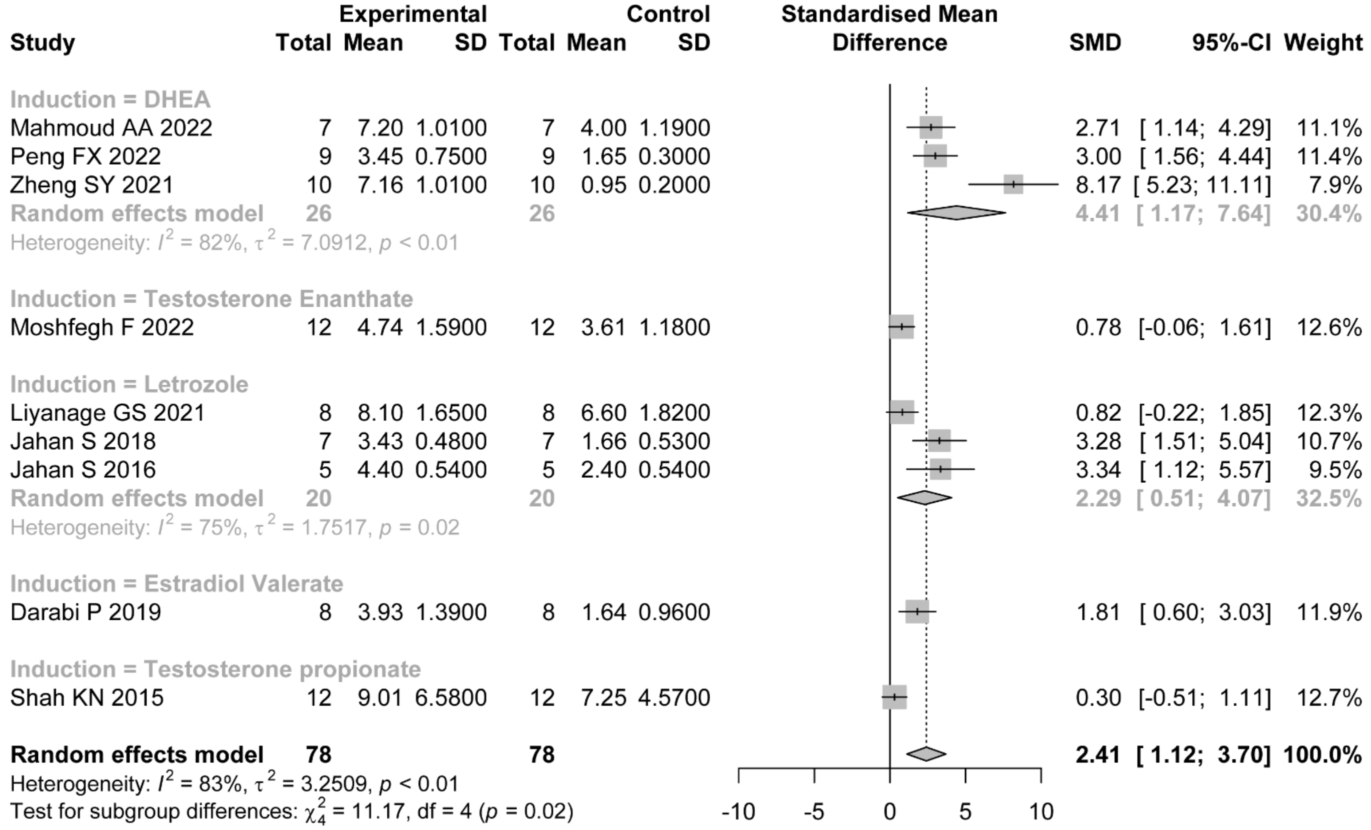

Figure S7 Subgroup analyses of the count of corpus luteum with **(A)** type of flavonoids, **(B)** dose, **(C)** duration of administration, and **(D)** PCOS induction drug.

Supplementary Figure 8

(A)

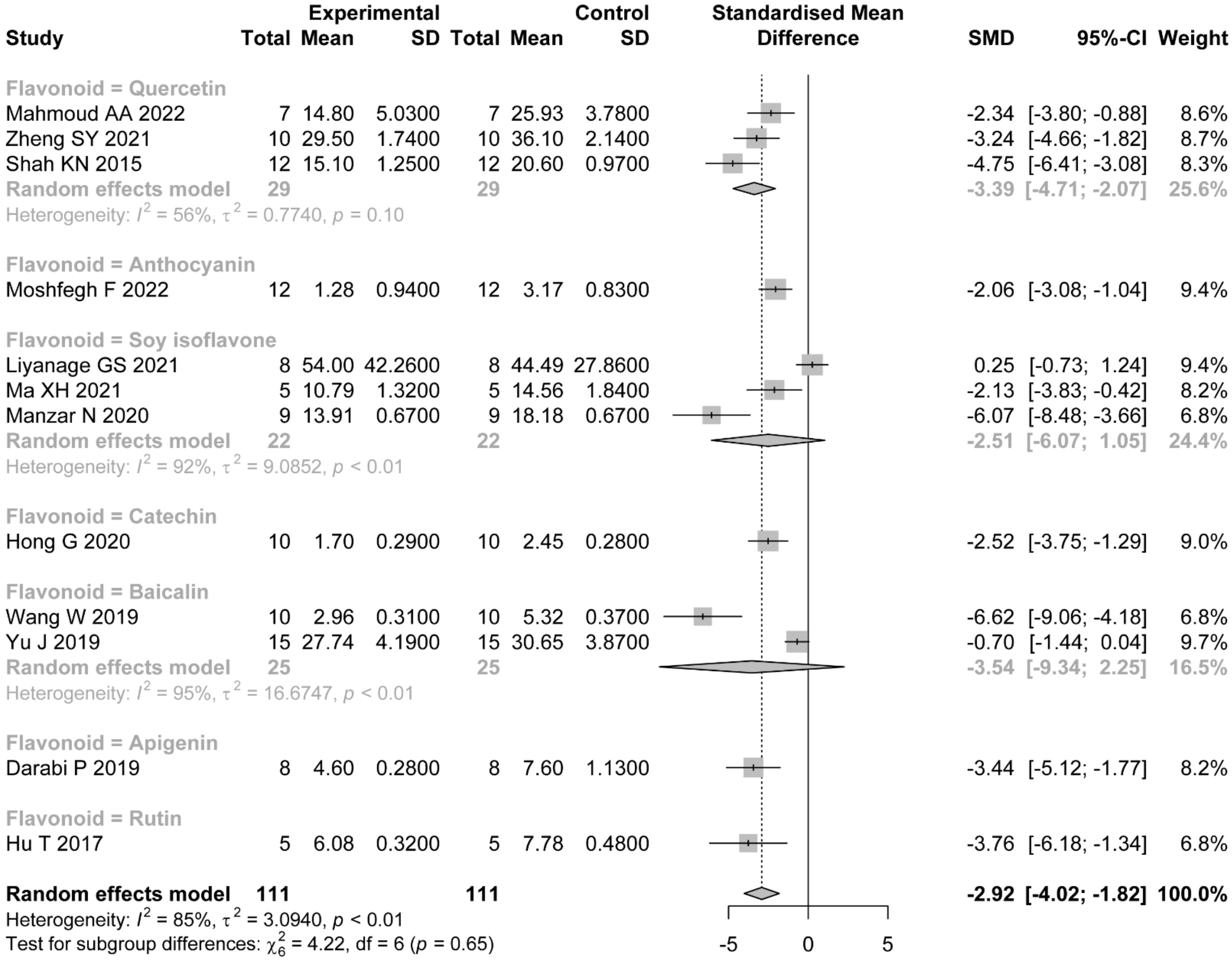

(B)

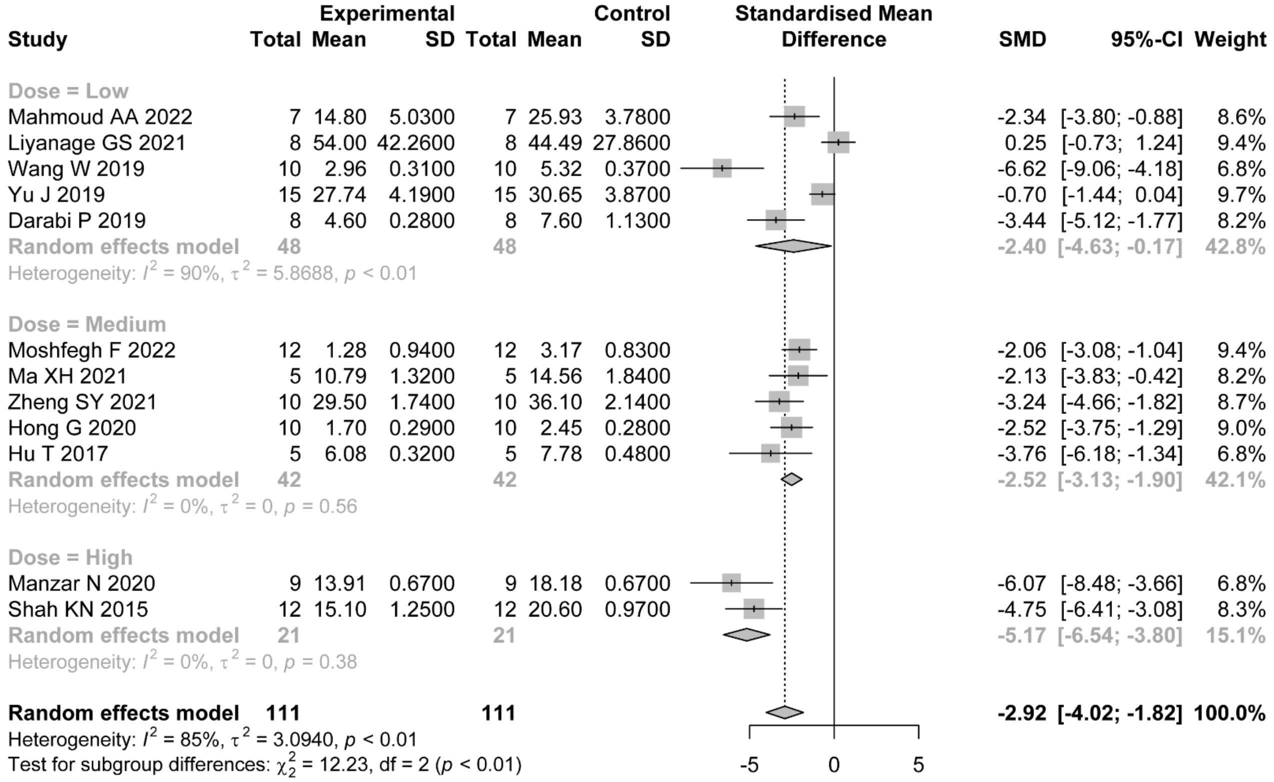

(C)

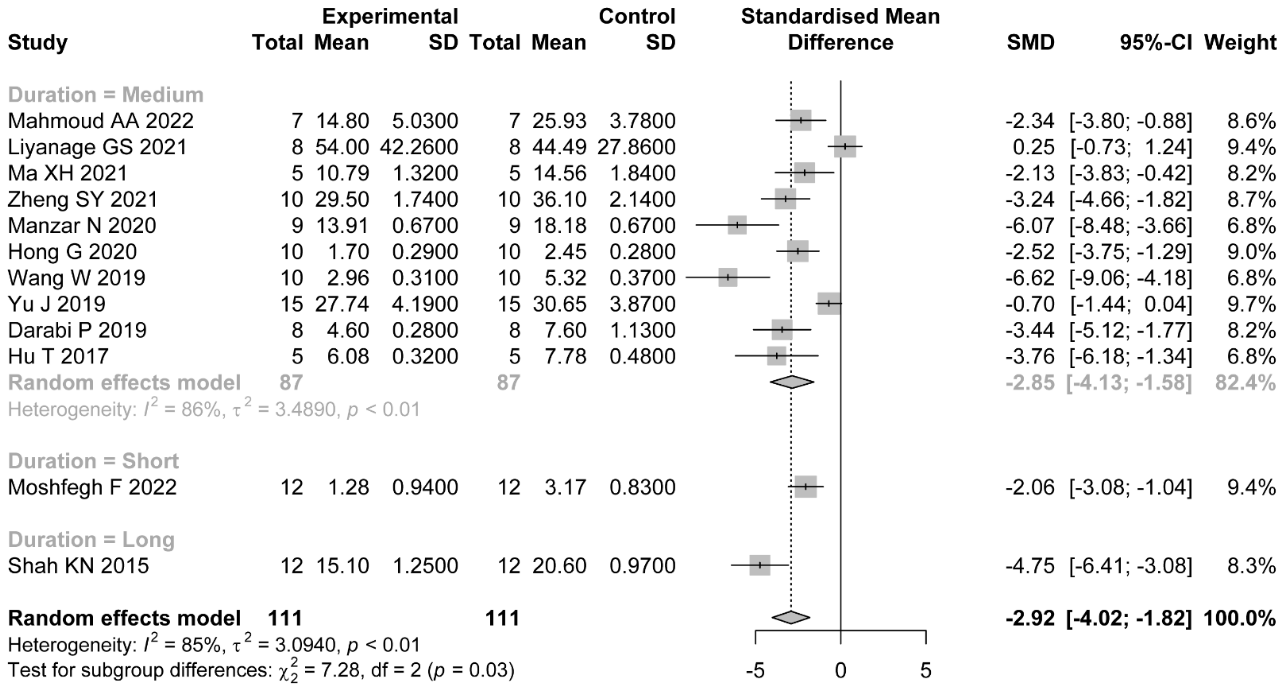

(D)

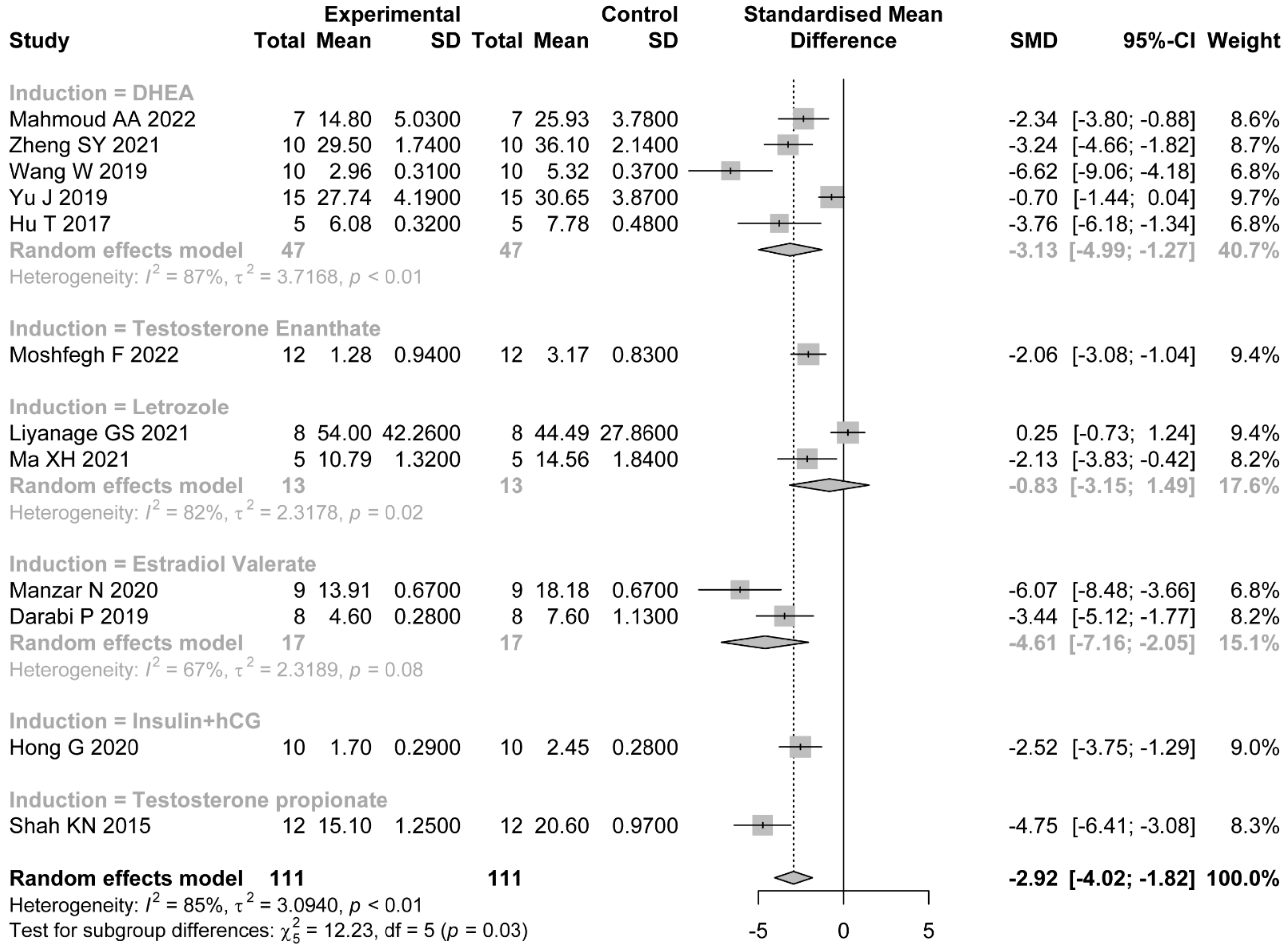

Supplementary S8 Subgroup analyses of LHwith **(A)** type of flavonoids, **(B)** dose, **(C)** duration of administration, and **(D)** PCOS induction drug.

Supplementary Figure 9

(A)

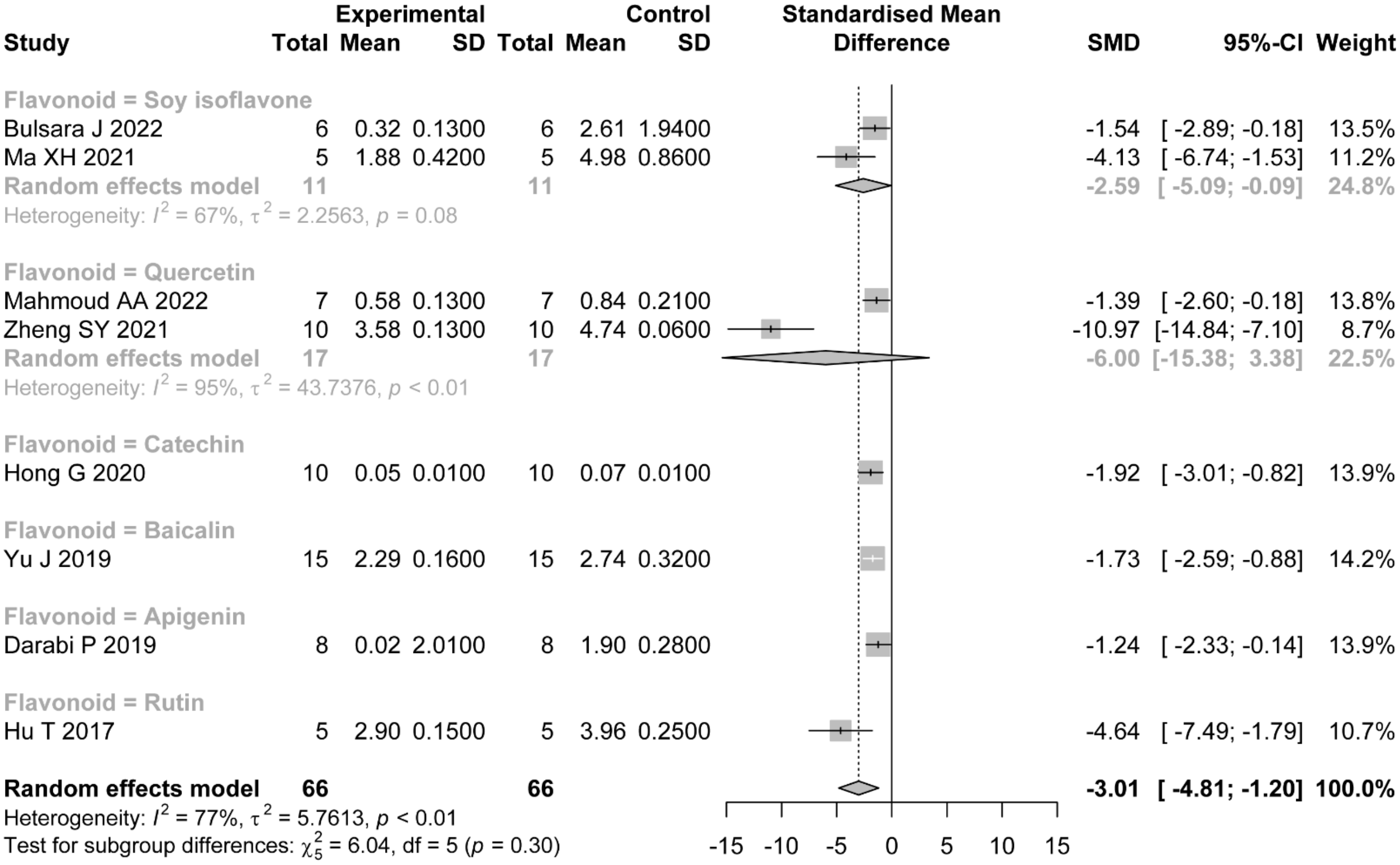

(B)

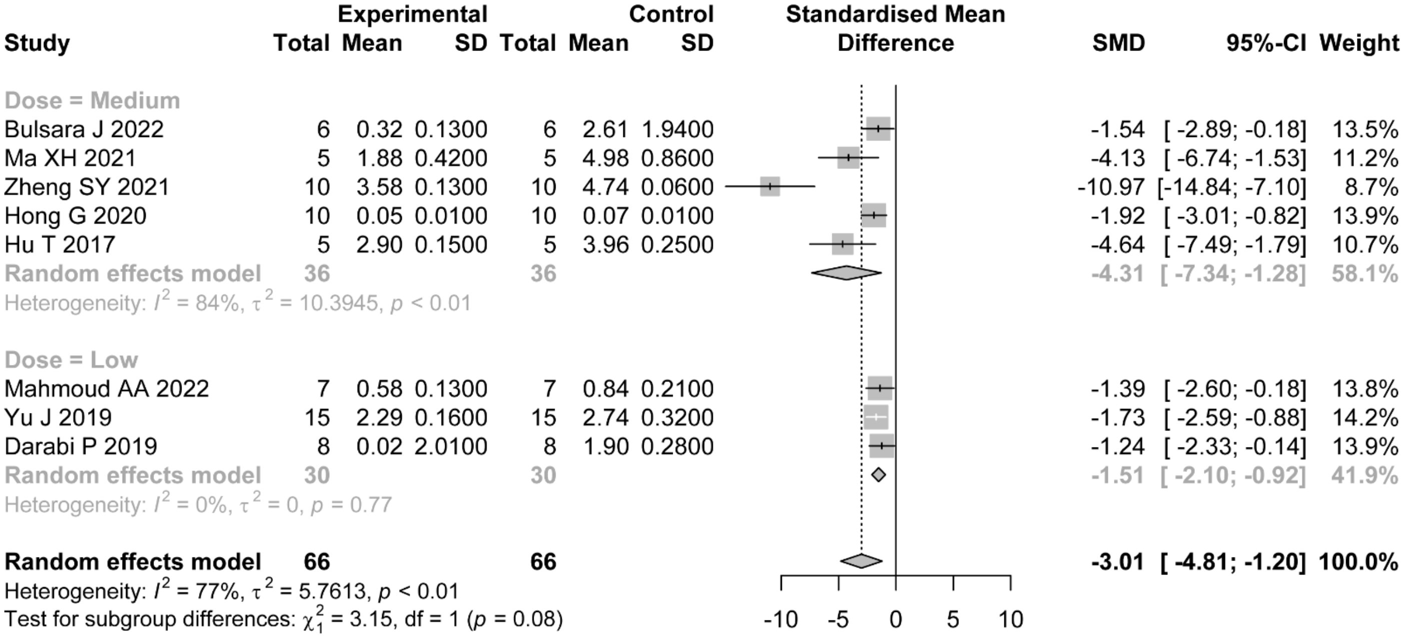

(C)

Study	Experimental Total	Experimental Mean	Experimental SD	Control Total	Control Mean	Control SD	SMD	95%-CI	Weight
**Duration = Short**									
Bulsara J 2022	6	0.32	0.1300	6	2.61	1.9400	-1.54	[-2.89; -0.18]	13.5%
**Duration = Medium**									
Mahmoud AA 2022	7	0.58	0.1300	7	0.84	0.2100	-1.39	[-2.60; -0.18]	13.8%
Ma XH 2021	5	1.88	0.4200	5	4.98	0.8600	-4.13	[-6.74; -1.53]	11.2%
Zheng SY 2021	10	3.58	0.1300	10	4.74	0.0600	-10.97	[-14.84; -7.10]	8.7%
Hong G 2020	10	0.05	0.0100	10	0.07	0.0100	-1.92	[-3.01; -0.82]	13.9%
Yu J 2019	15	2.29	0.1600	15	2.74	0.3200	-1.73	[-2.59; -0.88]	14.2%
Darabi P 2019	8	0.02	2.0100	8	1.90	0.2800	-1.24	[-2.33; -0.14]	13.9%
Hu T 2017	5	2.90	0.1500	5	3.96	0.2500	-4.64	[-7.49; -1.79]	10.7%
Random effects model	60			60			-3.31	[-5.46; -1.16]	86.5%
Heterogeneity: $I^2 = 80\%$, $\tau^2 = 7.2887$, $p < 0.01$									
**Random effects model**	**66**			**66**			**-3.01**	**[-4.81; -1.20]**	**100.0%**

Heterogeneity: $I^2 = 77\%$, $\tau^2 = 5.7613$, $p < 0.01$
Test for subgroup differences: $\chi^2_1 = 1.88$, df = 1 ($p = 0.17$)

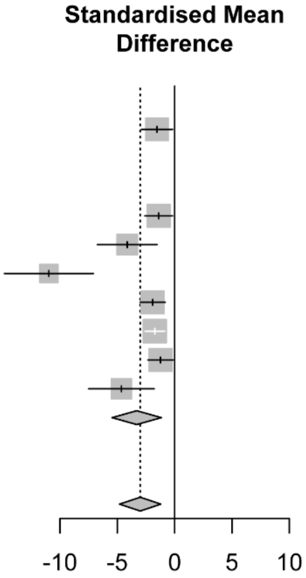

(D)

Study	Experimental Total	Experimental Mean	Experimental SD	Control Total	Control Mean	Control SD	SMD	95%-CI	Weight
**Induction = Letrozole**									
Bulsara J 2022	6	0.32	0.1300	6	2.61	1.9400	-1.54	[-2.89; -0.18]	13.5%
Ma XH 2021	5	1.88	0.4200	5	4.98	0.8600	-4.13	[-6.74; -1.53]	11.2%
Random effects model	11			11			-2.59	[-5.09; -0.09]	24.8%
Heterogeneity: $I^2 = 67\%$, $\tau^2 = 2.2563$, $p = 0.08$									
**Induction = DHEA**									
Mahmoud AA 2022	7	0.58	0.1300	7	0.84	0.2100	-1.39	[-2.60; -0.18]	13.8%
Zheng SY 2021	10	3.58	0.1300	10	4.74	0.0600	-10.97	[-14.84; -7.10]	8.7%
Yu J 2019	15	2.29	0.1600	15	2.74	0.3200	-1.73	[-2.59; -0.88]	14.2%
Hu T 2017	5	2.90	0.1500	5	3.96	0.2500	-4.64	[-7.49; -1.79]	10.7%
Random effects model	37			37			-4.38	[-8.47; -0.29]	47.4%
Heterogeneity: $I^2 = 88\%$, $\tau^2 = 15.8993$, $p < 0.01$									
**Induction = Insulin+hCG**									
Hong G 2020	10	0.05	0.0100	10	0.07	0.0100	-1.92	[-3.01; -0.82]	13.9%
**Induction = Estradiol Valerate**									
Darabi P 2019	8	0.02	2.0100	8	1.90	0.2800	-1.24	[-2.33; -0.14]	13.9%
**Random effects model**	**66**			**66**			**-3.01**	**[-4.81; -1.20]**	**100.0%**

Heterogeneity: $I^2 = 77\%$, $\tau^2 = 5.7613$, $p < 0.01$
Test for subgroup differences: $\chi^2_3 = 2.94$, df = 3 ($p = 0.40$)

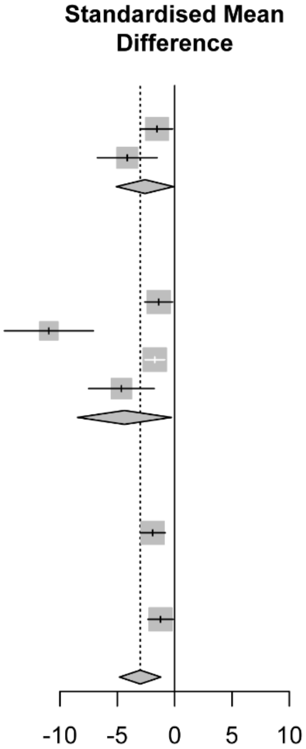

Figure S9 Subgroup analyses of LH/FSH with **(A)** type of flavonoids, **(B)** dose, **(C)** duration of administration, and **(D)** PCOS induction drug.

Supplementary Figure 10

(A)

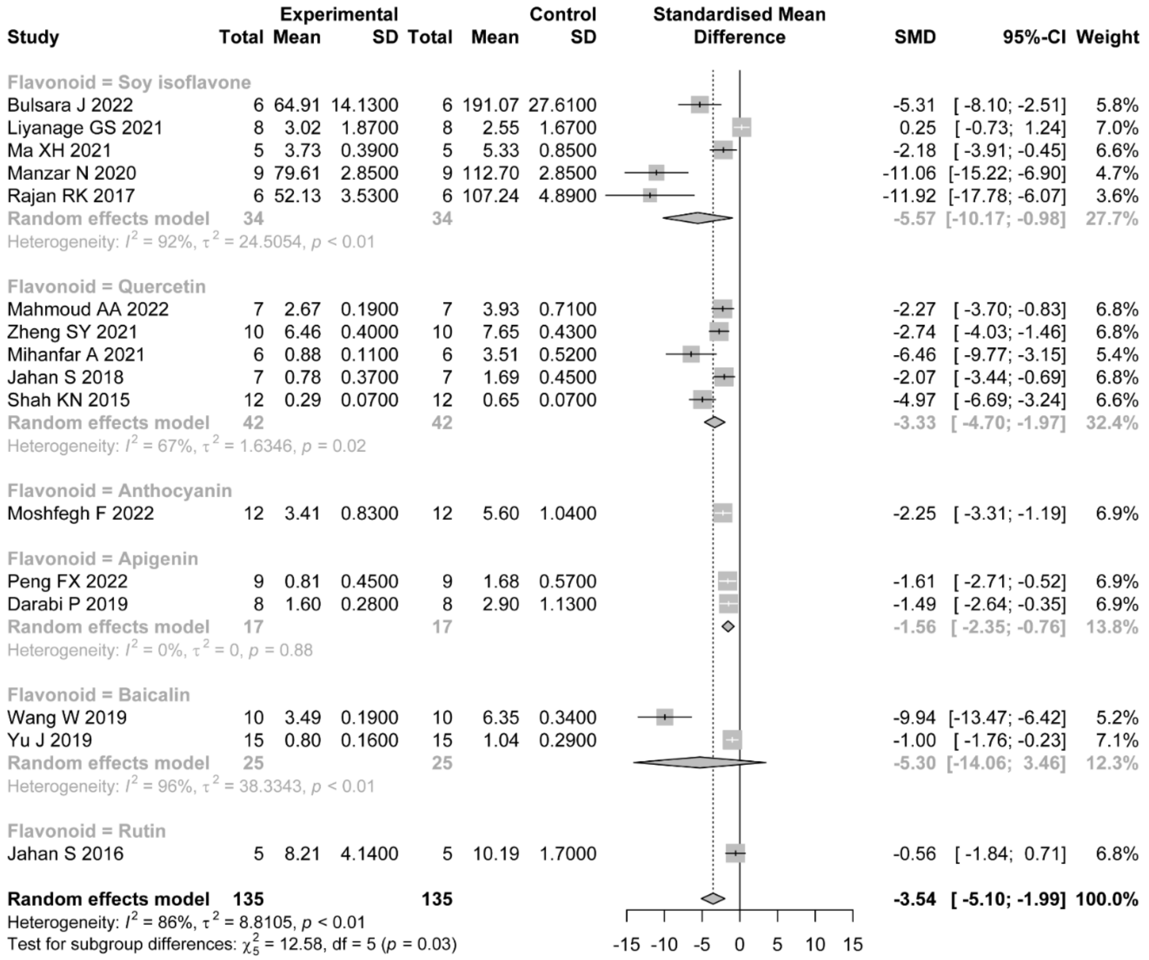

(B)

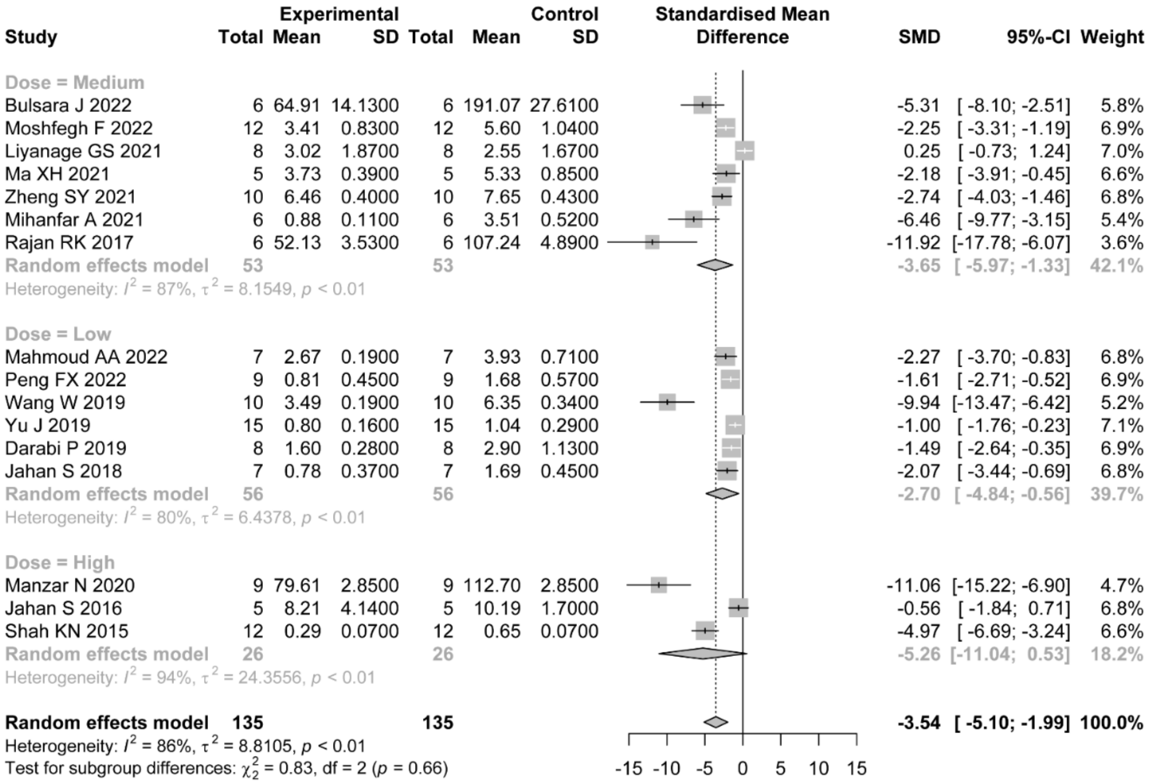

(C)

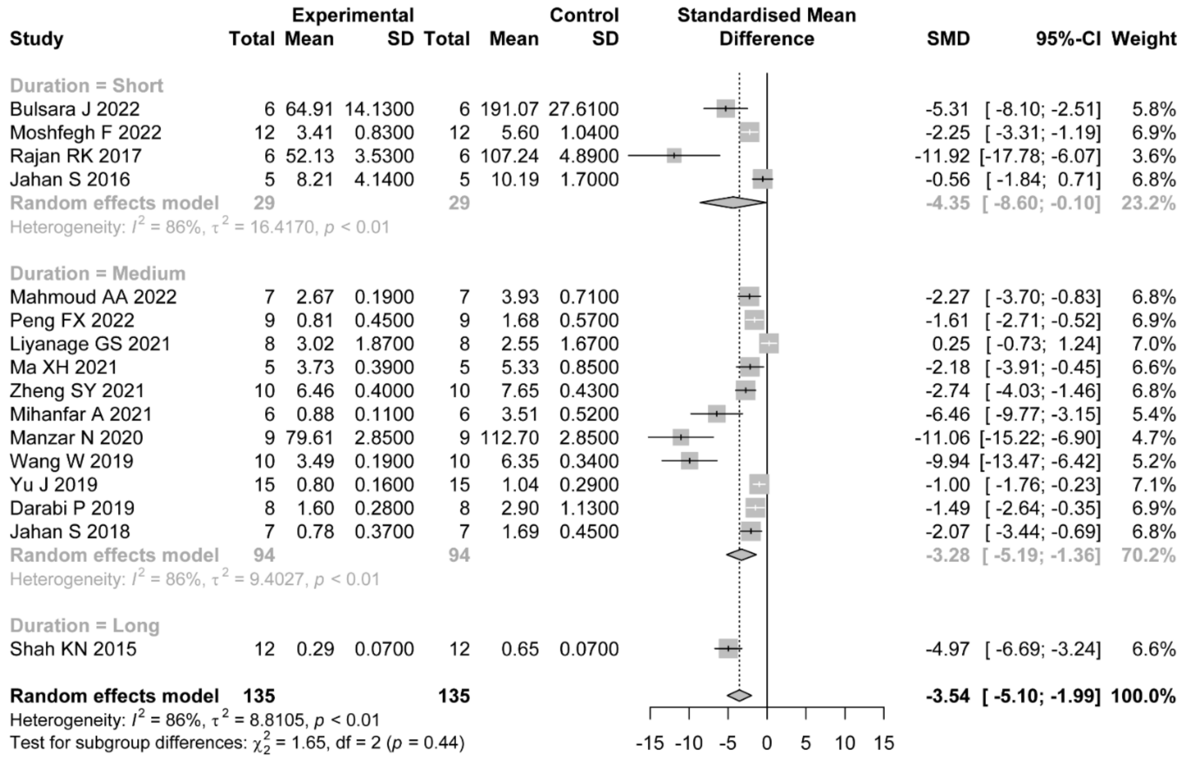

(D)

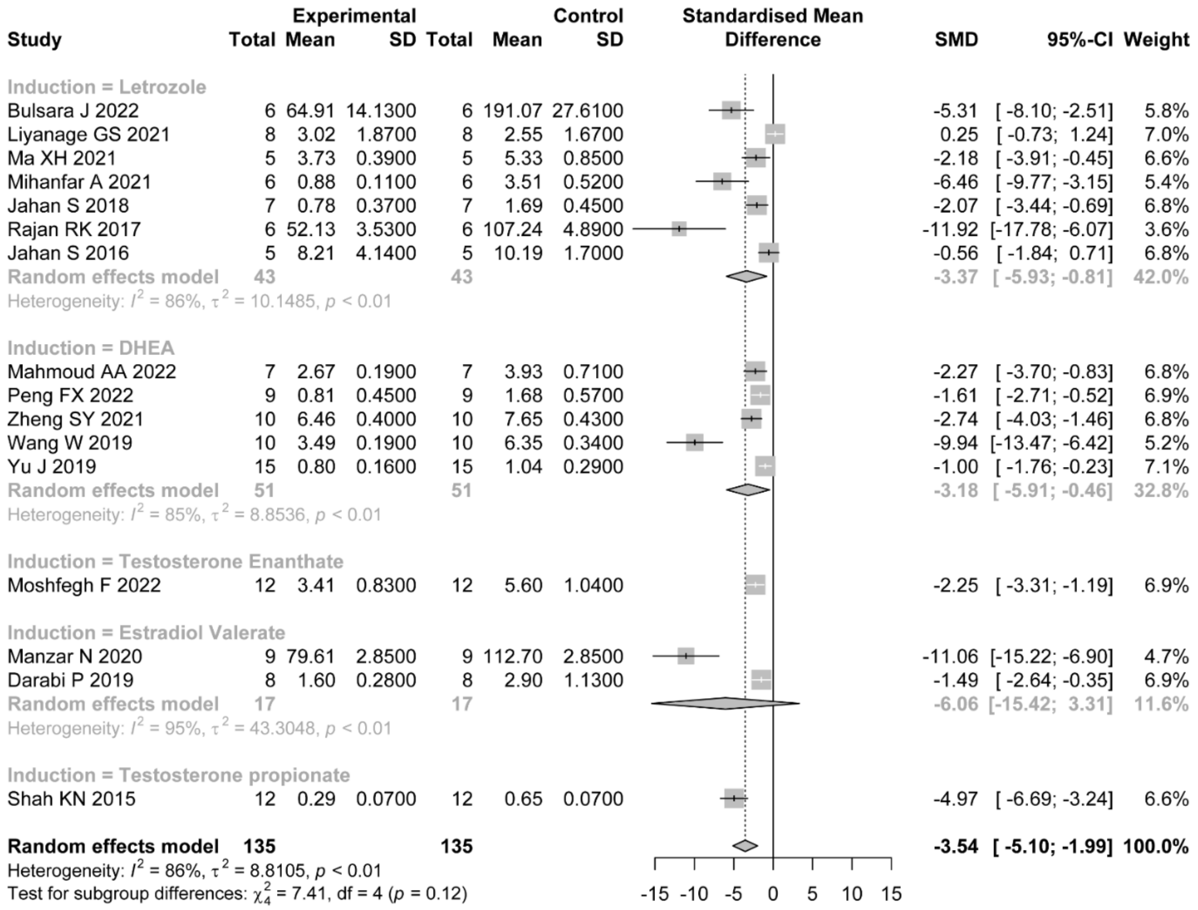

Figure S10 Subgroup analyses of FT with **(A)** type of flavonoids, **(B)** dose, **(C)** duration of administration, and **(D)** PCOS induction drug.

Supplementary Figure 11

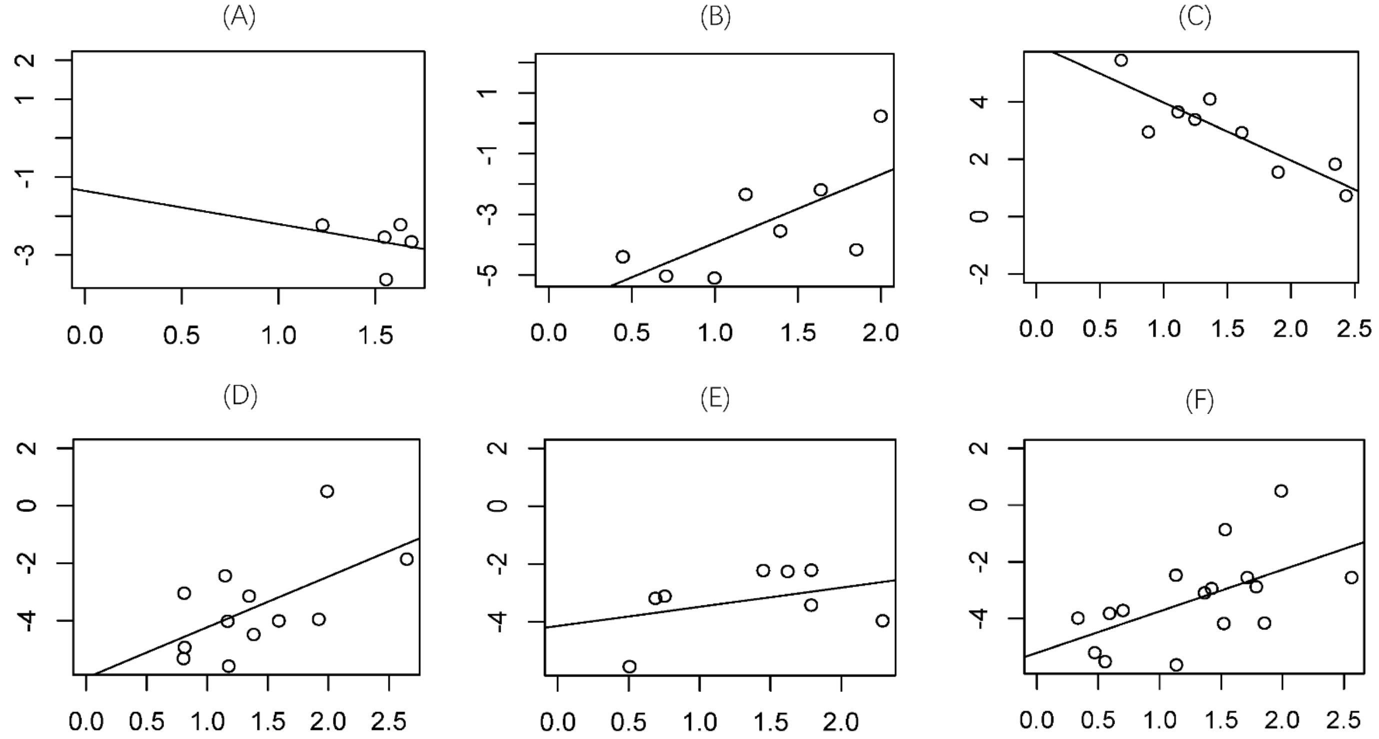

Figure S11 Egger's bias test of (A) the count of atretic follicles, (B) the count of cystic follicles, (C) the count of corpus luteum, (D) LH, (E) LH/FSH, and (F) FT.

Supplementary Figure 12

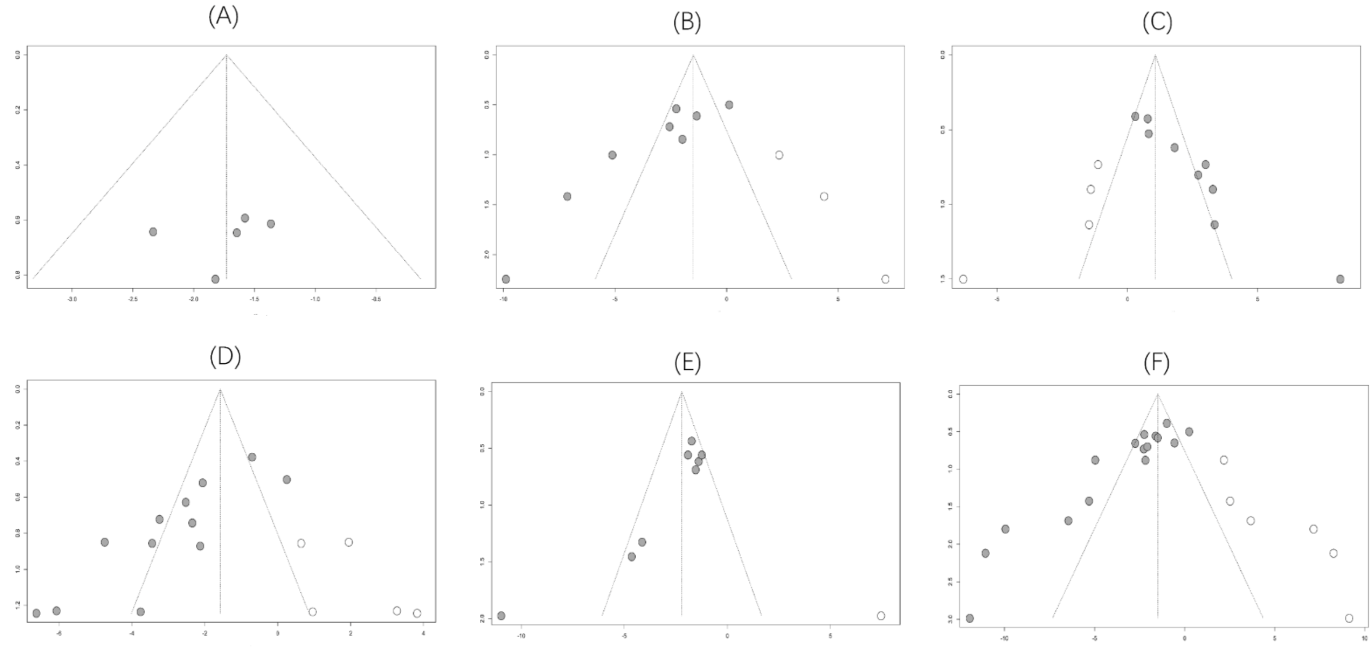

Figure S12 Trim-and-fill evaluation of (A) the count of atretic follicles, (B) the count of cystic follicles, (C) the count of corpus luteum, (D) LH, (E) LH/FSH, and (F) FT.

Supplementary Figure 13

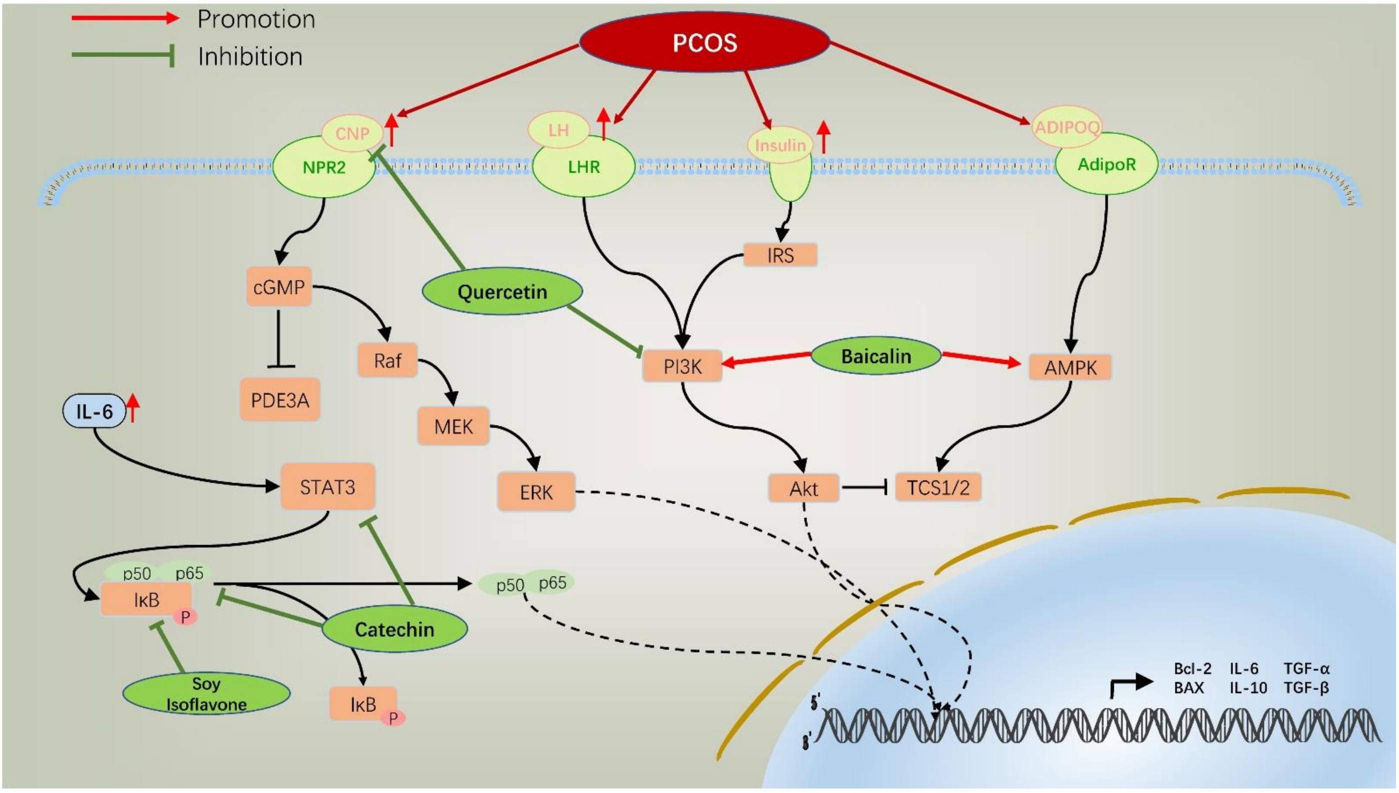

Figure S13 Possible mechanisms of flavonoids on PCOS animal models.
